# Assessments of Medical Student's Knowledge About Radiation Protection and Different Imaging Modalities in Jeddah, Saudi Arabia

**DOI:** 10.1155/ijbi/1528291

**Published:** 2025-04-24

**Authors:** Raghad Aljondi, Rahaf Alem, Rowa Aljondi, Abdulrahman Tajaldeen, Salem Saeed Alghamdi, Mohammed Majdi Toras

**Affiliations:** ^1^College of Medicine, King Abdulaziz University, Jeddah, Saudi Arabia; ^2^Department of Applied Radiologic Technology, College of Applied Medical Sciences, University of Jeddah, Jeddah, Saudi Arabia; ^3^Department of Family Medicine, King Fahad Armed Forces Hospital, Jeddah, Saudi Arabia

**Keywords:** imaging modalities, knowledge, medical students, radiation, radiation protection, radiology

## Abstract

**Introduction:** Doctors can play a significant role in attributing to patient safety concerning exposure to ionizing radiation. Therefore, healthcare professionals should have adequate knowledge about radiation risk and protection of different medical imaging examinations. This study aims to evaluate the knowledge about radiation protection (RP) and applications of different imaging modalities (IMs) among medical students in their clinical years and intern, in Jeddah, Saudi Arabia.

**Materials and Methods:** A cross-sectional study based on an online questionnaire was performed in Jeddah, Saudi Arabia, on 170 medical students during January 2024; the study participants included clinical years medical students (from Years 4 to 6) and interns of both gender and basic year medical students, and specialists and consultants were excluded. For each participant, the percentage of correct answers was calculated for the knowledge RP and knowledge in IMs separately, and each participant will have two scores, RP knowledge score (RPKS) and IM knowledge score (IMKS).

**Results:** A total of 170 medical students responded and completed the questionnaire. The overall levels of awareness and knowledge of the students was determined through calculations of their scores in answering the questionnaire; students in this study group have low average knowledge score in RP, which is 43, while they have moderate–high knowledge score in IMs, which is 68. Regarding the knowledge score, for the RPKS, the best participant scored 82, while the worst scored 0, whereas for IMKS, the best participant score 100, while the worst scored 0. However, according to the SD, participants generally differ between each other by 19 in RPKS and 31 in IMKS.

**Conclusions:** The assessments of medical students' knowledge regarding radiation exposure in diagnostic modalities reveal a low level of confidence in their knowledge of ionizing radiation dose parameters. Furthermore, the mean scores on overall knowledge assessments indicate a need for improvement in RP knowledge for medical students. To address this gap, a comprehensive modification of the undergraduate medical curriculum's radiology component is required by enhancing active learning approaches and integrating radiation safety courses early in the medical curriculum. Medical education institutions could implement ongoing workshops, online modules, and certification programs to reinforce radiation safety principles.

## 1. Introduction

Recently, the number of radiological examination that involved exposure to ionizing radiation has been increased worldwide [1, 2]. These radiological examinations are requested by physicians and performed routinely due to their usefulness in disease diagnosis and providing treatment plan [3]. The radiation dose received from some imaging modalities (IMs) such as computed tomography and x-ray machine increases stochastic effects for patients and the lifetime risk of cancer [4–6]. These risks of radiation can be minimized by understanding and applying the principle of radiation protection (RP) in the field of diagnostic medical exposure [7]. Therefore, healthcare professionals should have adequate knowledge about radiation risk and protection of different medical imaging examinations.

Several studies have assessed the physician's knowledge about ionizing radiation dose and exposure risks from varying specialties, and they demonstrated suboptimal results [8–12]. A study in Riyadh, Saudi Arabia, revealed that most physicians had lacked knowledge about the radiation dose of common radiological examinations, with 58% of their participants underestimating the radiation dose [8]. Another study conducted in Jeddah, Saudi Arabia, included consultants, specialists, residents, physicians, and emergency doctors, found a poor knowledge about radiation doses and risks among doctors working in emergency department [11]. Although research regarding the medical students' knowledge of ionizing radiation and its associated risk have been previously studied [13–19], there is a paucity of studies that have examined the specific knowledge of medical students about radiation risk and safety towards different IMs [20, 21].

As the majority of radiological examinations are requested by physicians, they should have the basic knowledge of radiation dose and the risk and benefit from different IMs. This study aims to evaluate the knowledge about RP and applications of different IMs among medical students in their clinical years and internship, in Jeddah, Saudi Arabia.

## 2. Materials and Methods

A cross-sectional study based on an online questionnaire performed in Jeddah, Saudi Arabia, on 170 medical students during January 2024. The study participants included clinical years medical students (from Years 4 to 6) and interns of both gender and basic years medical students, and specialists and consultants were excluded. To ensure statistical adequacy, we conducted a G∗Power analysis (version 3.1.9.7) software, with Type II error set as 0.05, test power set as 0.80, the ratio of males/females set as 0.42 (expected), ratio of non-Saudi/Saudi as 0.11, ratio of private/governmental institute as 0.25, and ratio of no lecture/lecture as 0.43. The effect size was set as Cohen's *d* = 0.5 in case of *t*-test and Cohen's *f* = 0.25 in case of ANOVA. The maximum suggested sample size was 180. This indicated that a sample size of 170 would provide sufficient power to detect meaningful differences/effects. Additionally, our sample size aligns with similar studies in the field [14, 20], suggesting that our findings are comparable and generalizable within the studied population.

A modified validated questionnaire of previously published studies in a multiple-choice format test was used to assess the knowledge of medical students about RP and different IMs [14, 20, 21]. The final questionnaire was then tested and reviewed by a panel of experts in the field of education and radiology from the University of Jeddah, Saudi Arabia. These experts assessed the clarity, relevance, and comprehensiveness of the items, providing feedback on potential ambiguities or redundancies. They addressed concerns about the content validity of the questionnaire by adding or removing items to ensure comprehensive coverage of the basic knowledge of radiation dose and the risk and benefit from different IMs, ensuring that the questions were relevant to the targeted population of medical students and avoiding double-barreled questions.

The multiple-choice survey was categorized into three sections: general information (7 items), RP (11 items), and IMs (9 items). The questionnaire consisted of two main parts measuring knowledge: knowledge in RP and knowledge about IMs. Answers to the questions were either correct or incorrect. Any correct answer was coded as 1, while incorrect as 0. In addition, the questionnaire contained a question that if the students have taken any lectures regarding RP as part of their medical study. It also included oriented demographic questions such as age, gender, university, and in which clinical year they are. The confidentiality of personal information was protected throughout the study by keeping the participants' information anonymous. On the first page of the survey, each participant gave their electronic informed permission. All of the participants were requested to be completely honest when answering the questions. This survey's participation was entirely voluntary and without remuneration. The datasets generated and analyzed during the current study are available from the corresponding author on reasonable request.

Data were entered and analyzed utilizing Software Package for Social Science (SPSS) statistical software, version 26. Demographic variables were summarized using frequency distribution. Quantitative data were expressed as mean, standard deviation (SD), minimum, and maximum. Numbers and percentages represent qualitative data. The main statistical tools used in the analysis were the independent sample *T*-test and the one-way ANOVA. These tests were used to assess whether demographic groups, that is, gender (males/females) differs significantly in their overall knowledge scores for RP and IM. Independent sample *T*-tests are used when there are two categories in the demographic variable (example: gender has two categories, namely, male and female). Methods like one-way ANOVA tests are used when the categories are more than 2 such as group of students. Because we were comparing the group's means, the *T*-test and ANOVA were the most appropriate methods. For each participant, the percentage of correct answers was calculated for the knowledge in RP and knowledge in IMs separately, and each participant will have two scores, RP knowledge score (RPKS) and IM knowledge score (IMKS). A *p* value < 0.05 was considered a significant result.

## 3. Results

A total of 170 medical students responded and completed the questionnaire representing the population in this study. Among these students, 63 (37.1%) were male and 107 (62.9%) were female. The sample characteristics and demographics of the participants are presented in Table 1. Most of the participants (71%) are in the 4th or 5th clinical year (41% + 30%, respectively). The majority of the sample participants (96%) are Saudi Arabian. Then, 152 (89%) of the participants study at governmental universities while 18 (11%) study at private universities. Furthermore, most of the participants (79%) had previous lecture(s) in RP. In terms of participant self-evaluation of knowledge in RP, the majority (86%) is roughly divided between “moderate” and “high,” while in terms of the self-evaluation of knowledge in IM, most of them (71%) chose “high.”

The percentage of correct answers for questions about RP is shown in Table 2. Most of the sample participants (i.e., > 70%) were knowledgeable about the following questions: *The type of tissue that is most sensitive to radiation, the age group that is most sensitive to ionizing radiation, and the method used to reduce the amount of exposure to ionizing radiation*. Around the half or slightly above (48%–58%) of the medical students were knowledgeable about the following questions: *What the dosimeter device is used for, the material used for the protection against radiation risks, and the principle of the International Commission on Radiological Protection (ICRP) for as low as reasonably achievable (ALARA)*. Most of the participants (60% and more) do not have knoweldge about the following questions: *The disease that could be a result of stochastic effects due to ionizing radiation, the maximum permissible occupational effective dose limit for radiation workers per year, the effective whole body dose limits for a patient per year, how far one should stand from the* x-ray *source during the radiological-guided procedure without using any protection, and the approximate effective radiation dose from postero-anterior chest* x-ray.

Table 3 shows the percentage of correct answers for questions about IM. The majority of the participants (> 60%) were knowledgeable about the right answer for most of the listed questions. Under two questions only, almost half of the participants (47%–53%) were knowledgeable about the following questions: *The IMs that use x-rays, and the procedures involving the greatest radiation exposure to the patient.*

The overall levels of awareness and knowledge of the students are represented in Table 4. As shown in the table, students in this study group have low average knowledge score in RP, which is 43, while they have moderate-high knowledge score in IM, which is 68. Regarding the knowledge score, for the RPKS, the best participant scored 82, while the worst scored 0, whereas for IMKS, the best participant scored 100, while the worst scored 0. However, according to the SD, participants generally differ between each other by 19 in RPKS and 31 in IMKS.

Concerning the relation between the knowledge score and demographic variables, the demographic variables for each of RPKS and IMKS are shown in Tables 5 and 6, respectively.

The differences in knowledge scores between gender, universities, clinical year, and previous lectures were compared, and the mean RPKS is significantly different between the two types of universities (governmental/private), with a *p* value of 0.001 (which is < 0.05). This significance is clearly reflected in the mean RPKS for the two groups: 45 and 30, respectively. As a result, students in the governmental universities have relatively better knowledge in RP. In addition, the mean RPKS is also significantly different (*p* value = 0.0001, which is < 0.05) between those who have taken a lecture in RP and those who have not. This significance is clearly reflected in the mean RPKS for the two groups: 47 and 30, respectively. Consequently, students who have already taken a lecture in RP have relatively better knowledge in RP than those who have not. Conversely, other characteristic variables (gender, nationality, and clinical year) are not significantly associated with RPKS, where the *p* values are > 0.05, under all the three variables (Table 5).

There are three significant values (*p* value < 0.05) presented in Table 6 which express the association between IMKS and the characteristic variables (university type, clinical year, and previous lecture in RP). Starting with the university type, the significance is clearly reflected in the mean IMKS of students in the governmental and private universities: 72 compared to 34, respectively. Therefore, students in governmental universities have better knowledge in IM.

Moreover, the significance is visibly reflected in the mean IMKS of students in different clinical years: 57 in the 4th year compared to slightly above 75 for the 5th and 6th years, while it is 66 for the intern. Subsequently, students in the 4th year have lower knowledge about IM than students in the 5–6th year. Furthermore, the significance is clearly reflected in the mean IMKS of students who have taken a lecture in RP and students who have not: 72 and 50, respectively. Accordingly, students who have already taken a lecture in IM have better knowledge than those who have not. In contrast, the other characteristic variables (gender and nationality) are not significantly associated with IMKS, where the *p* values are > 0.05.

## 4. Discussion

The main findings of the present study are that medical students have overall low to medium levels of awareness, attitude, and practice of RP and IMs with 48% and 68%, respectively. Students who had already taken previous lectures in radiology during clinical years in medicine had better knowledge in RP and IMs. The most significant improvement in IMKS was apparent between Years 5 and 6.

The present study demonstrates that most of the medical students had low average knowledge scores in RP. This may reflect the approaches of the academic staff towards issues associated with RP and need to improve their level in effective radiation dose for the patients, because defective knowledge and misconstruction of radiation doses may lead to prescription of unnecessarily ionizing imaging examinations, resulting in an increased risk for patients. It is also apparent that this deficiency of knowledge will make it difficult to notify patients about the risks and benefits of a radiological examination [14, 18, 22].

The distance between x-ray sources is important for medical students' safety. The effects of small level exposure to ionizing radiation are of concern to a huge number of people including employees receiving radiation exposure on work [23–27]. Our study revealed that most of the participants do not have knoweldge about how far one should stand from the x-ray source. The results of this study was found to be in line to several studies which had documented insufficiencies in knowledge among medical students and doctors about either consideration of ionizing radiation or the usage of involved equipment [28–32]. For improving knowledge among medical students about hazards of radiation, an active education would be effective to distribute information to those who have inadequate knowledge about radiology and RP [33]. Moreover, medical education institutions could provide ongoing workshops, online modules, and certification programs to reinforce radiation safety principles throughout medical training and early clinical practice.

RP courses for medical students have to include organic effects of radiation, validations of exposure, process optimization, benefit–risk analysis, and characteristic doses for each type of examination. A study from Saudi Arabia revealed that there was a significant correlation between the knowledge score and attendance at training courses on RP with (*p* ≤ 0.01), with the individuals who attended training sessions more frequently benefiting from such interaction [22]. Moreover, it is unexpected that a substantial number of participants did not attend radiation safety training sessions or refresher courses, as evidenced by their knowledge score [22]. International Commission on Radiological Protection (ICRP) suggests a total of 5–10 h for RP education and training for medical students [34]. Additionally, awareness of the advantages and disadvantages of the usage of ionizing radiation in medicine must be a part of RP education and training for medical students [35]. Integrating comprehensive RP education into medical training can address deficiencies in knowledge regarding radiation safety. A study highlighted that only 10.3% of medical students attended a RP course, with significant differences in knowledge levels across clinical years [20]. This suggests that early and continuous education on radiation safety is crucial.

Medical students start taking radiology modules in their curriculum in 4th year of clinical practice. However, their knowledge about radiology was limited. This finding is in line with previous studies that assessed medical students' education about radiation and its effects [13, 17, 22, 24, 36]. These studies conclude that most medical students have limited awareness about radiation sources, hazards, and safety as well as misunderstandings about risk of exposure existing among medical students that could potentially affect healthcare outcomes. Allocating specific objectives regarding radiation in the curriculum along with practical radiology ward rotation for medical students are essential that can improve performances regarding health principles and approaches within the communities. In addition, active learning methodologies have been shown to be more effective than passive approaches in teaching radiology-related content. A systematic review and meta-analysis found that active teaching methods significantly improved medical students' radiology knowledge and skills [37]. Incorporating interactive sessions, simulations, and case-based learning can enhance understanding and retention of RP principles.

Enhancing medical students' knowledge of radiation safety directly impacts patient safety. Educated practitioners are better equipped to make informed decisions about IMs, minimizing unnecessary radiation exposure to patients [38]. This study highlights numerous critical gaps in medical students' understanding of critical RP components, which should be considered when creating an undergraduate medical curriculum that is prepared for future difficulties [13, 21]. A broad-based introduction to radiation science within medical curricula has been advocated to bridge existing knowledge gaps and promote safe clinical practices.

### 4.1. Limitations and Advantages

This research is limited due to the possibility of bias presented in the responses as our data relies on participants' self-assessment; there is a possibility of overestimation or underestimation of their actual knowledge levels. Regardless of this attainable bias, the levels of knowledge were still insufficient among medical students; further research could be done on site by giving the questionnaire to students at the same class under their supervision. Additionally, the representativeness of our sample may be a limitation, as it is drawn from a specific region, and may not fully reflect the broader population of medical students. These factors should be considered when interpreting our findings. Despite the limited data of this study, it can be used to improve the current curriculum in medical universities by making new beneficial changes in the curriculum in order to enhance the knowledge of RP and different IMs with additional training courses that can boost the practical skills of students and furthermore the clinical physicians in the hospital. Future studies were recommended to incorporate objective assessments of knowledge, such as standardized tests, and include a more diverse sample from multiple institutions to enhance generalizability. This study could be intended to cover not only students but also medical physicians, dentists, nurses and other workers to demonstrate the total level of knowledge about RP and different IMs used in a hospital, taking into consideration clinicians who are directly or indirectly associated to the radiology field.

## 5. Conclusion

Our assessment of medical students' knowledge of radiation exposure in diagnostic imaging reveals a low level of confidence in their understanding of ionizing radiation dose parameters. These findings highlight the need for enhanced education and training to improve both competency and patient safety. Addressing this gap requires a comprehensive revision of the radiology component within the undergraduate medical curriculum, ensuring that students receive structured and effective instruction on RP and safe imaging practices. We recommend enhancing active learning approaches and integrating radiation safety courses early in the medical curriculum. These courses should comprehensively cover fundamental principles, dose estimation, and evidence-based strategies for minimizing radiation exposure. Additionally, medical education institutions could implement ongoing workshops, online modules, and certification programs to reinforce radiation safety principles throughout medical training and early clinical practice. This will ensure that future healthcare professionals are adequately prepared to make informed and evidence-based decisions regarding medical imaging, thereby enhancing both patient care and safety.

## Figures and Tables

**Table 1:** Sample characteristics and demographics of participants.

**Variable**	**Categories**	**N**	**%**
Gender	Male	63	37.1
	Female	107	62.9

Clinical year	4th	70	41.2
	5th	51	30.0
	6th	24	14.1
	Intern	25	14.7

Nationality	Saudi	164	96.5
	Non-Saudi	6	3.5

University type	Governmental	152	89.4
	Private	18	10.6

Previous lecture in RP	Yes	135	79.4
	No	35	20.6

Self-evaluation for knowledge in RP	Low	24	14.1
	Moderate	70	41.2
	High	76	44.7

Self-evaluation for knowledge in IM	Low	7	4.1
	Moderate	43	25.3
	High	120	70.6

**Table 2:** The percentage of correct answers for questions about RP.

**Question**	**% of correct answers**
Which type of the following tissues is most sensitive to radiation?	72%
What is the dosimeter device used for?	48%
Which of the following diseases could be a result of stochastic effects due to ionizing radiation?	39%
Which one of the following age groups is most sensitive to ionizing radiation?	74%
Which of the following materials are used for protection against radiation risks?	58%
Which of the following methods are used to reduce the amount of exposure to ionizing radiation?	75%
What is the maximum permissible occupational effective dose limit for radiation workers per year?	25%
What is the effective whole body dose limits for a patient per year?	11%
Which of the following principles of the International Commission on Radiological Protection (ICRP) for as low as reasonably achievable (ALARA)?	50%
How far should you stand from the x-ray source during the radiological-guided procedure without using any protection?	11%
What is the approximate effective radiation dose from postero-anterior chest x-ray?	16%

**Table 3:** The percentage of correct answers for questions about IM.

**Question**	**% of correct answers**
Which of the following imaging modalities do you think use x-rays:	47%
Which of the following medical imaging modalities that produce cross-sectional images:	80%
Which of the following medical imaging modalities has no radiation:	73%
Which of the following medical imaging modalities that uses high-frequency sound waves to create images of organs inside the body:	79%
Which of the most commonly used medical imaging modalities that uses ionizing radiation to produce images:	68%
Which of the following medical imaging modalities that uses a radioactive material to produce images of the internal structure:	60%
Which of the following procedures involves the greatest radiation exposure to the patient:	53%
In which of the followings medical imaging modalities that using a metal device is contraindication:	71%
Which of the following imaging modalities is safe for diagnosis of pregnant women:	79%

**Table 4:** The overall levels of awareness, attitude, and practice.

**Score**	**Min**	**Max**	**Mean**	**SD**
RPKS	0.00	82	43	19
IMKS	0.00	100	68	31

**Table 5:** RPKS vs. demographic/characteristic variables.

**Characteristic variable**	**Categories**	**N**	**Mean**	**SD**	**p** ** value**
Gender	Male	63	43.0	19.5	0.802
	Female	107	43.8	18.5	

Nationality	Saudi	164	43.4	18.7	0.794
	Non-Saudi	6	45.5	22.3	

University type	Governmental	152	45.1	18.7	**0.001**
	Private	18	29.8	13.5	

Clinical year	4th	70	42.1	18.8	0.237
	5th	51	47.2	19.0	
	6th	24	44.7	18.6	
	Intern	25	38.5	18.0	
	Total	170	43.5	18.8	

Previous lecture in Radiology	Yes	135	47.1	17.7	**0.0001**
	No	35	29.6	16.7	

**Table 6:** IMKS vs. demographic/characteristic variables.

**Characteristic variable**	**Categories**	**N**	**Mean**	**SD**	**p** ** value**
Gender	Male	63	70.5	30.3	0.361
	Female	107	66.0	31.6	

Nationality	Saudi	164	67.2	31.1	0.270
	Non-Saudi	6	81.5	29.5	

University type	Governmental	152	71.7	28.9	**0.0001**
	Private	18	34.0	29.1	

Clinical year	4th	70	57.4	32.7	**0.001**
	5th	51	78.9	24.7	
	6th	24	75.9	25.7	
	Intern	25	65.8	34.5	
	Total	170	67.7	31.1	

Previous lecture in Radiology	Yes	135	72.4	30.9	**0.0001**
	No	35	49.5	24.6	

## Data Availability

The data that support the findings of this study are available on request from the corresponding author. The data are not publicly available due to privacy or ethical restrictions.
